# Space closure using aligners

**DOI:** 10.1590/2177-6709.25.4.085-100.sar

**Published:** 2020

**Authors:** Ricardo Martins Machado

**Affiliations:** 1 Universidade Federal do Rio de Janeiro (Rio de Janeiro/RJ, Brazil).

**Keywords:** Esthetic aligners, Invisalign, Removable orthodontic appliances, Clear dental appliances

## Abstract

**Introduction:**

Due to the search for more aesthetic and comfortable alternatives to perform orthodontic treatments and to the great technological development, orthodontic aligners have assumed great importance. More and more complex treatments have been carried out with these appliances without, however, having all aspects involved in their use being studied in depth. Its biomechanical planning requires different approaches than those used in fixed orthodontics, as the force systems involved in movements, responses and side effects are distinct, and the professional must be prepared when opting for the technique.

**Objective:**

The objective of this article is to perform an evaluation of the force systems created on the space closure with aligners, its characteristics, and problems, as well as make some suggestions to overcome the difficulties inherent to its use.

**Conclusion:**

Space closure with aligners is possible, but depends on the correct selection of the patient, in addition to requiring the proper planning of the applied forces. The use of auxiliary resources and overcorrections to address the deficiencies of the aligner systems should always be considered. Digital planning should be used as a map of the force systems that will be applied, and not just as a marketing tool, keeping in mind that determining the objectives and the way to achieve them is the responsibility of the orthodontist, and that treatment plans must be individualized for each situation, following appropriate biomechanical precepts.

## RESUMO

**Introdução:**

Devido à busca por alternativas mais estéticas e confortáveis para realizar tratamentos ortodônticos e ao grande desenvolvimento tecnológico, os alinhadores ortodônticos assumiram uma grande importância. Tratamentos cada vez mais complexos vêm sendo realizados com esses dispositivos sem que, no entanto, sejam estudados em profundidade todos os aspectos envolvidos na sua utilização. Seu planejamento biomecânico requer abordagens diferentes da Ortodontia fixa, pois os sistemas de forças envolvidos nas movimentações, respostas e efeitos colaterais são distintos, e o profissional deve estar preparado ao optar por essa técnica.

**Objetivo:**

O objetivo deste artigo é fazer uma avaliação dos sistemas de forças envolvidos no fechamento de espaços com alinhadores, suas características e problemas, bem como apresentar algumas sugestões para contornar as dificuldades decorrentes de seu uso.

**Conclusão:**

O fechamento de espaços com alinhadores é possível, mas depende da correta seleção do paciente, além de exigir o adequado planejamento das forças aplicadas. O uso de recursos auxiliares e sobrecorreções para suprir as deficiências dos sistemas de alinhadores deve ser sempre considerado. O planejamento digital deve ser usado como um mapa dos sistemas de forças que serão aplicados, e não apenas como ferramenta de marketing, tendo em mente que a determinação dos objetivos e da maneira de atingi-los é de responsabilidade do ortodontista e que os planejamentos devem ser individualizados para cada situação, seguindo preceitos biomecânicos adequados.

## INTRODUCTION

Since the first vacuum-formed plastic orthodontic device for dental alignment was proposed by Kesling^1^, there has been a great advance in the possibilities of treatment with this type of appliance. This idea was put aside for many years until it was taken up by Ponitz,^2^ in 1971, who suggested the making of vacuum appliances made with transparent material on plaster setups. Following a similar principle, McNamara et al^3^ suggested tooth movement with aligners; and in 1993, Sheridan^4^ introduced the Essix system, which uses the technique of bubbles and bumps, created with reliefs and deformations in the models, made with heated special pliers, to move teeth. In 1997, two Stanford MBA students, without any dental training, applied their knowledge in computing and CAD/CAM technology to develop and launch the Invisalign system of aligners based on digital technology. Since its creation, encouraged by the success of Invisalign and the easier access to this kind of technology, several other aligner systems have been created by other companies, using the same principle. The current market presents a huge variety of digitally produced systems, with great appeal to the public. 

Plastic aligners have great esthetic advantage, more comfort and make it a lot easier to keep a good oral hygiene, when compared with traditional fixed appliances,^5^ with ease of feeding and chewing being the most highlighted qualities referred on studies^6^. Previously having it’s use restricted to simple movements to align incisors, the plastic aligners have been broadening its applications and are now used to treat almost every kind of malocclusion, including more complex cases, with good esthetic and functional outcomes.^7^ In many situations aligners can be as efficient as fixed appliances, even though in other cases they still lack some improvement, like torque control or proper occlusal settlement.^8^

In this scene, the companies that produce those aligners have been funding lots of researches for development of new materials and technologies to supply the orthodontists needs. The great evolution of software for digital planning allied with the use of artificial intelligence and more sophisticated algorithms allow more precise and predictable outcomes of the force systems generated by these devices, proposing more reliable solutions.

**Figure 1:**
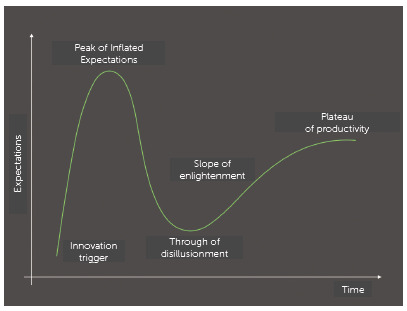
Gartner’s Hype Cycle for new technologies.^6^

In the introduction of any new technology a sequence of events can be observed. The first phase is the trigger of the innovation, when it appears on the market, presented as the best solution for all problems. Seduced by all the positive aspects massively highlighted by the developers, professionals start to try to use it in some situations. The use of the technology experiments a dramatic increase. The manufacturing companies then start to invest more and more in publicity. Stories of success begin to pop up on various fronts, making other professionals feel confident to start using it as well, hoping for wonderful outcomes. This phase is called peak of inflated expectations. As it is used without thorough evaluation and concern to the restrictions in its indications of use, failures to achieve the expected outcomes begin to be reported, because the limits of the technique are still uncertain. At this point, a feeling of disillusionment begins to set in and many abandon its use. It is called the through of disillusionment. We then enter a slope of enlightenment, where more scientific studies and trials are made, bringing better understanding of the actual pros and cons of the technique and, after a period of maturation where the limitations and methods are better defined, the technique finds its place among the tools of regular use by the professionals, who will be able to use it in all its potential on the proper situations, taking the necessary precautions to achieve the best results. This phase is called the plateau of productivity. This sequence of events is known as the Gartner’s Hype Cycle for new technologies,^9^ as shown in Figure 1.

This situation can be verified in the adoption of the aligners. The planning of orthodontic movements must be made differently if compared to fixed orthodontics, and the desired and undesired effects of each force system will depend on other factors. Because of that, orthodontists with traditional background on fixed orthodontics will need to adapt, if they wish to use the aligners and achieve results as they are used to. Thus, the objective of this article is to perform an evaluation of the force systems created on the space closure with aligners, its characteristics and problems, as well as make some suggestions to overcome the difficulties inherent to its use.

## PATIENT SELECTION AND ORTHODONTIST’S ATTITUDE

Regardless of all the high technology behind clear aligners, the most important criteria for success in the treatments falls under properly choosing the patient. Some clinical conditions, such as dental open bites, are more prone to be successfully treated by aligners, while others, like deep bites associated to spacing, are more difficult to be treated, but above it all, the orthodontist must be able to correctly evaluate the psychological and behavioral profile of the patient, to identify the degree of engagement and motivation. Since aligners are removable appliances that need to be worn continuously, the treatment demands high level of discipline and commitment to achieve the objectives planned. A perfect biomechanical planning and all the technology involved have no use if the patient is not adherent to the treatment and the aligners are not correctly used. It is very important that the communication between orthodontist and patient is extremely clear, and that the patient take co-responsibility for the success of the treatment, considering that a great part of it depends on that.

Maybe this last issue is the main reason why orthodontist resist to adopt aligners as a routine option, since they consider having less control over the results, when compared to fixed appliances, which depend less of patient collaboration.

The orthodontist who decides to start using aligners must have in mind that, besides having to motivate the patient during the treatment, will have to take a more proactive attitude while planning, anticipating the possible side effects of the chosen biomechanics. Differently from the fixed appliances, where they have the possibility to be more reactive and correct it at each visit, depending on the response to activations made on the previous appointment, on the treatment with aligners the orthodontist has the activations pre-determined and all the compensations must be created before the movements are made. For this reason, it is of utmost importance a deep knowledge of the system characteristics and all the effects of intended biomechanics. Just as individualization on bracket placement and archwire sequences on fixed appliances according to the objectives of the treatment, with aligners we should be able to clearly visualize where to go and how to fulfil each step of the treatment to correctly prescribe the movements and auxiliary resources, as well as understand the limitations of each case. That is why, despite the smaller chair time during treatment, the time invested in the construction of the treatment plan tends to be bigger and demands great dedication.

## DIGITAL SETUPS

The first dental movements made with aligners were made over physical setups or small sequential modifications on plaster models, on which the aligners were vacuum-pressed. Activations could be done also by special pliers causing controlled deformations on the aligners to create pressure points that would cause the desired tooth movement. These techniques were very laborious and had very little precision. The introduction of the digital models in orthodontics had a very important role in the dissemination of aligners. Its precision and accuracy have already been proved in many studies^10^ ^,^ ^11^ and they have been gradually replacing the plaster models. Every treatment with aligners is based on movements made on digital models, that are divided in stages by software specifically designed for that purpose, which is made respecting the physical properties of the material of which the aligner is made and the limits of the biological response of the patient. The planning systems are becoming more sophisticated, using the huge databases created by the initial, follow-up and final records of millions of cases treated worldwide, to harvest lots of information over the tooth movements and the responses to activations. Using artificial intelligence and machine learning resources to treat these data and feed the algorithms, the treatment plans provided are becoming more and more reliable.

One of the greatest indirect advantages of the massification of the aligners use was the dissemination of the use of digital setups, imperative for their manufacturing. Different companies have different resources to perform aligners staging, and this interface became a high value asset for the elaboration of the treatment plans and to the communication between the orthodontist and the team that produces the aligners, but it can also be used, in many situations, for fixed orthodontics cases, like in trays for indirect bonding, for instance. The digital setups can also be used to improve the communication between orthodontist and patient, providing a way to visualize the treatment goals and its phases. But is this last one the best way to face these softwares? As simply a marketing and communication tool? When orthodontists take that attitude, one of the greatest powers of this tool, which is the possibility of constructing a detailed map of all the force system that will be applied during the treatment and the anticipation of its effects, gets set aside. The possibility to test in a practical and fast way many treatment possibilities, makes the orthodontist's choice of one treatment plan over another more conscious and safe. This visual analysis, paired with the clinical experience of the clinician, allows the planning of overcorrections, preparations, compensations, and anticipation of undesired side effects that may occur as consequence of the chosen biomechanics. By doing this, the digital treatment plan helps to minimize errors and makes treatments safer and more precise.

By the deep knowledge of the biomechanics characteristics of the appliances and dental movements, one can use many resources to achieve the planned outcomes. The results observed on aligner treatments are improving by their association with auxiliary tools, like elastics, skeletal anchorage, binaries with elastics and even the use of brackets in some segments of the arch - the hybrid treatments. By correctly using these tools, it is possible to overcome some of the limitations of the aligners and, according to the learning curve of each professional, optimize treatments and improve the predictability of the planned movements, because the undesired side effects will be reduced.

## PECULIARITIES OF ALIGNER’S BIOMECHANICS

**Figure 2:**
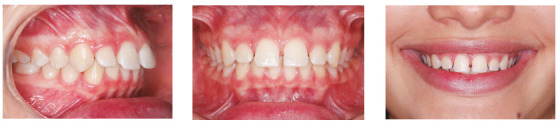
Patient presenting diastemas with exaggerated overbite, good posterior intercuspation and good incisors exposure - example of a situation where what seems to be simple for planning with fixed orthodontics becomes a complex treatment to be performed with aligners, due to the limitations of the technique.

When putting together a force system for any orthodontic movement, a series of factors have to be taken into consideration, such as: the point of application of the force, the force magnitude, the velocity of application, it’s direction, the duration and the effects it will produce.^12^ These questions are only some that can emerge and, when treating with aligners, will have different answers, if compared to fixed appliances. An example of these differences can be seen in Figure 2, which shows a clinical case considered simple for treatment with fixed orthodontics, but that represents a great challenge to be treated with aligners. The patient had good posterior intercuspation, diastemas in the maxillary arch, accentuated overbite and good incisors exposure. The vertical control and control of buccal-lingual inclination of the incisors during the anterior retraction for space closure is a great difficulty of the aligner systems, as it causes lingual inclination of the incisors, increasing the overbite and incisors exposure. In a case like this, the results of treatment with aligners would be very unfavorable and difficult to achieve.

## POINT OF APPLICATION

**Figure 3:**
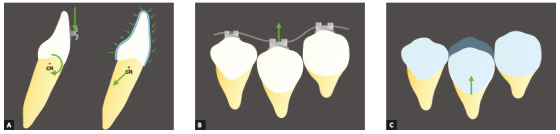
Examples of the difference in points of application of forces between fixed appliances and plastic aligners during intrusion (A). The brackets and wires system push teeth toward the wire (B) while aligners pull the teeth to the desired direction (C).

Instead of having the force applied to one single point at the buccal or lingual surface of the tooth, as happens in fixed orthodontics, there will be a plastic surface embracing the whole crown of the tooth (Fig 3).

The decomposition of forces must take into consideration all the tooth surface to determine the resulting force on that system. Besides that, on fixed appliances, the wire is tied to the brackets and delivers the forces by pulling or pushing the teeth (Fig 3B), while with aligners, where there is no fixed structure connecting the appliance and the tooth, the force is delivered by the contact of the plastic with the crown, pulling it to the desired position (Fig 3C).

Because of this characteristics, dental crown anatomy will have a great impact on the response of some tooth movements. Teeth with short expulsive crown shapes, that will have less contact surface with the plastic of the aligner, tend to express some movements less efficiently than teeth with larger and more retentive crowns. For this reason extrusion is an example of a unfavorable movement to be done with aligners,^13^ while it’s a simple movement to be done with fixed appliances. Canines rotation is another movement with very low predictability and must be overcorrected.^14^

## ATTACHMENTS

The attachments are resources normally used to address this issue. By adding little amounts of composite with specific designs to specific areas of the crown, the dental anatomy is changed to improve the retainability and create more favorable shapes and contact surfaces to deliver the desired force. This allows that these movements occur more effectively and predictably. Unfortunately, these resources present a negative effect as well, especially in the anterior region of the dental arch, because it worsens the esthetics of the aligners, making it even worse than the esthetics of ceramic appliances.^15^

**Figure 4:**
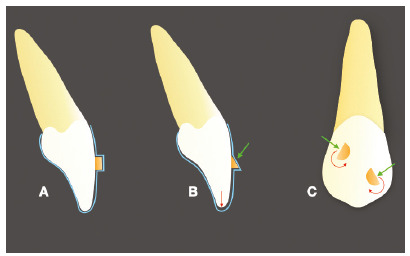
Examples of attachments: (A) passive or retention, (B) optimized for extrusion and (C) optimized for root inclination. The optimized attachments show active surfaces for specific movements.

**Figure 5:**
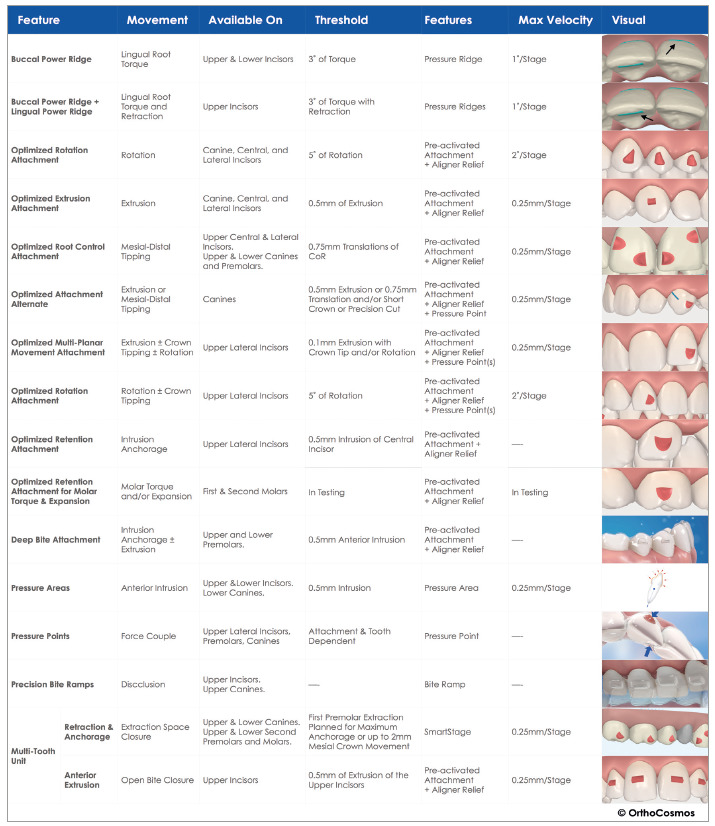
Examples of the great variety of attachments and resources existing in the Invisalign system, which is only one of the many options available.^16^

Attachments may function as retention auxiliaries, whose only intention is to keep the aligner in place, or they can be active, when the contact of the plastic with the tooth surface is supposed to deliver some force component in a specific direction. In this case, attachments have plan surfaces positioned in a way that favors the application of these forces. There is a great variety of shapes and sizes of attachments, according to each manufacturer, tooth anatomy and movement intended, as can be observed on Figures 4 and 5.

The proper selection of attachments may be a decisive factor on the predictability of the treatment, even though they are not indispensable to tooth movements in most cases. When the orthodontist chooses a specific aligner system, the algorithms in the software will have internally predefined parameters to, depending on the movements needed, suggest which attachments to use. However, this selection won’t always follow the same line of thought of the orthodontist, and may prioritize different movements from the ones desired to fulfill the planned outcomes. Those algorithms work according to a certain hierarchy of movements that will determine the automatic selection of attachments, normally based on the difficulty of the movement, and not necessarily on its relevance to the final results. If the case have, for instance, a tooth that needs to be intruded and rotated, the software will prioritize the rotation and suggest one attachment that favors the rotation over the intrusion, because the rotation is a more difficult movement to be done, even if the intrusion is a more important movement to the resolution of the main problem. This is where the orthodontist must have an active role in the attachment selection, and not only passively accept the suggestions given by the system. In many cases, it is necessary to change the attachments’ design, dimension and position to get the desired force system.

Another factor that must be taken into consideration is the attachment building technique. They must have an excellent adaptation to be able to work properly. There are variations in size and shape of the attachment templates and the active aligners, so replacement of lost attachments or adjustments to worn out ones must be done on the provided template, never directly on the aligner. Another factor that should be taken into consideration is the proper attachment placement technique. The templates must be perfectly adapted, and the slots completely filled with the composite of choice, but without excesses, because it could prevent the aligner to seat properly in place, affecting the movement intended. If there are excesses, it is important that they are completely removed before placing the aligners on.

## TREATING CASES WITH EXTRACTIONS

The boundaries of aligners treatments without teeth extractions is similar to the ones with fixed orthodontics. Severe crowding over 6 millimeters will probably cause great incisors protrusion and need significative expansion of the dental arches,^17^ which may compromise the stability of the results as well as the patient’s periodontal health in the long run. In these cases, it may be recommended to work with teeth extractions, normally first premolars, which can be challenging when the clinician decides to work with aligners.^18^ Other examples of cases that will have to deal with space closure are the ones with other extractions and surgically assisted palatal expansion (SARPE), where a great diastema is formed on the anterior portion of the upper arch.

It is very common to find in the literature case reports of successful cases of extractions where premolars have been removed because of severe crowding, but these cases normally do not need much retraction of the anterior teeth. The torque control of the incisors during retraction, a critical point in any retraction, even with fixed appliances, poses an even tougher challenge in aligner therapy, due to its physical properties. Some studies suggested that side effects of treatments with extractions, more specifically the tipping of the teeth adjacent to the extraction spaces, should be corrected with fixed appliances, what would considerably increase treatment time.^19^

When considering space closure, some possibilities may be present. The space closure can happen with: maximum anchorage, where all the space will be consumed with anterior retraction; reciprocating movement, where part of the space will be used for anterior retraction and the rest of it will be closed by mesial movement of the posterior segment; or it can be closed mostly with mesial movement of posterior teeth without any anterior retraction, just by solving some anterior crowding, for example. On the next paragraphs, we will analyze the first and second situations.

## 1 - CASES OF MAXIMUM ANCHORAGE CONTROL

On the situations where the space closure must be done exclusively by retraction of anterior teeth, the clinician must take extra care. The anchorage control must be planned thoroughly and the use of resources such as miniplates or mini-screws should be taken into consideration as a valuable ally. They would help not only in the sagittal direction, but also help control the vertical movements. If the clinician chooses not to use those resources, he should be even more careful.

The elastic properties of clear aligners, similar to what would happen if, with fixed appliances, anterior retraction was made on a thin NiTi wire, would generate a clockwise force moment in the anterior part of the arch that would cause the incisors to incline lingually and extrude. The middle part of the arch will receive intrusive force components, that will tend to intrude the premolar and cause the molar to tip forward, due to the counterclockwise force moment in the posterior segment. With the extrusion of the incisors, interferences are created in the anterior area and a posterior open bite is set.

**Figure 6:**
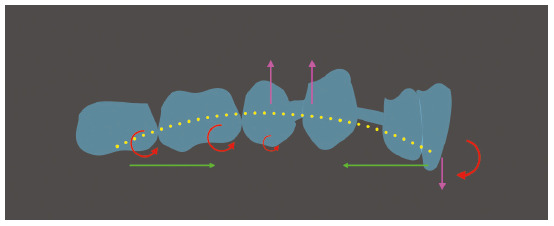
Force diagram showing the bow effect that happens during the anterior segment retraction with aligners, causing intrusion of the middle segment, mesial inclination of molars and extrusion with lingual tipping of the incisors.

This happen because the plastic will suffer horizontal deformation, like a wooden arch whose tips are connected by a wire and pulled towards each other. The fact that, due to the extraction site, the aligner has a segment without tooth support, it is even more prone to deflect (Fig 6).

As a result, if no action is taken to prevent that, the overbite will increase considerably while an open bite is settled in the posterior segment. When working with fixed appliances, similar effects could happen if the retraction was made with thin elastic wires, and the solution would be working with thicker and stiffer wires, to avoid the bow arch effect. With aligners, the material is the same through all the phases of treatment, so the only resource left to be used is to vary the way the forces are applied. With this in mind, the dentist can use some of the following strategies:

### a) Add curves of Spee

Following the same logic used in fixed appliances, one can plan a reverse curve of Spee on the lower arch and an exaggerated curve of Spee on the upper arch during the movements. This is made by planning some extrusion of the premolars, buccal inclination of the incisors’ crowns, intrusion of the incisors and distal tipping of the molars.

Since an intrusive effect will be planned for the incisors, the aligners won’t tend to lose tracking on those teeth; but in the middle section of the arch, where the premolars will be the vertical anchorage for the incisors movement, the extrusive force might cause the aligners to lose grip, damaging the expected results. Besides being the anchorage, some extra extrusion will be planned to compensate the bow arch effect. As discussed before, extrusive movements are difficult to be made by aligners because of the expulsive shape of teeth crowns. On this matter, the use of attachments can considerably improve the retention of the aligners and allow the movements to happen as planned.

Another possibility that can be adopted alone or combined with the attachments, depending on the tooth anatomy, is to use a vertical intermaxillary elastic on the maxillary and mandibular premolar, over bonded buttons. The extrusive force of the elastics will oppose the intrusive force generated by the aligner, balancing the force system and keeping the aligner well adapted. Some authors recommend the use of bite ramps on the lingual surfaces of the maxillary incisors to help the intrusive effect on the mandibular incisors; but, during anterior retraction, where the lingual inclination of the incisors is already challenging, the occlusal contact on those bite ramps would generate a force applied lingually to the center of resistance of the incisors, that would cause a force moment that would make them tip lingually, worsening the final outcome.

### b) Use movement staging

To gain more control over movements, they can be divided in stages. For instance, we can alternate between periods of distalization and periods of pure extrusion of the canines during retraction, reducing the chance of tracking loss, because in between distal movements the aligner has the time to express the movement of crown *versus* root tipping and the vertical control. Taking a closer look at this approach, it mimics what happens in the interaction between the wire and the brackets during sliding mechanics. At first, a crown inclination will occur and the binding generated between the bracket and the wire will generate a force moment that will move the root and upright the canine during the time between activations. After a period of this alternation of movements, a bodily movement will be achieved. With aligners there will be first a tendency to tooth inclination and intrusion (due to the bow arch effect), but if this tooth is kept without a new activation for distalization, it will have time to express only the compensatory movement, while another segment of the arch can be activated.

The inclination control can also benefit from this alternation of active and inactive distalization periods. For that to happen, the use of attachments on the teeth to be moved will be of great help. When the first inclination occurs, the little unsettling that will take place inside the attachments pod of the aligner will create additional forces that will tend to upright the tooth. Some companies provide active attachments with this goal, but a similar effect can be achieved with regular attachments properly placed, since the elastic force of the aligner mismatch will make it active.

As said before, between the activations for distalization of the canines, we can work on other aspects of the movements in a synergistic way, like the intrusion and the buccal inclination of the incisors, the distal inclination of the molars, or, if that is the case, the resolution of anterior crowding that might be present. 

The professional can work dividing all the anterior retraction in periods of canine distalization combined with incisors intrusion and protraction, alternated with partial retraction of the incisors. This approach, depending on the needs of each case, could be associated with the use of intermaxillary elastics, which would provide more control of the undesired effects that may appear, therefore, making the movements more predictable.

#### Clinical case 1

**Figure 7:**
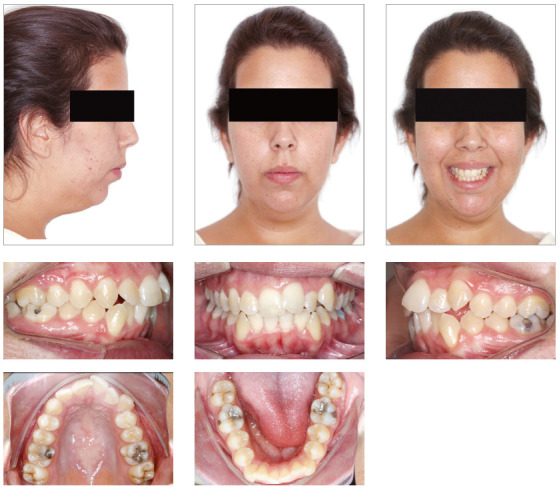
Female patient, 24 years of age, with bimaxillary protrusion and severe crowding on both arches, who chose to be treated with four first premolars extraction and compensation with aligners.

On Figure 7, we can see a patient with severe crowding on both arches, increased overjet, good molar relationship, mandibular deficiency, and vertical pattern. She had a 20% overbite and good periodontal health. The main indication was an orthognathic surgery with mandibular advance, which she refused. It was then decided to perform a compensatory treatment with aligners - a demand of the patient - with the extraction of the four first premolars. The space would be used for resolution of the crowding and incisors retraction on both maxillary and mandibular arches. Despite the weaker facial esthetic result, this was the option chosen by the patient, who refused any orthognathic surgical approach. 

**Figure 8:**
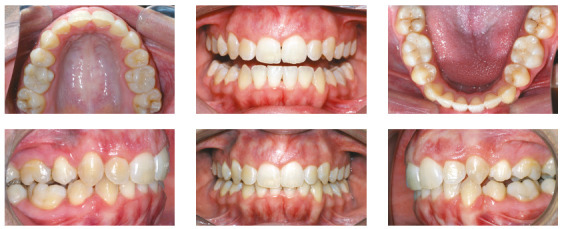
Situation at the end of the first aligners sequence. The curve of Spee was deepened with incisors extrusion and mesial inclination of molars and posterior open bite.

After a first set of 43 aligners, the patient, who was extremely compliant with the aligners use, had the extractions spaces closed, but with open bite on both sides on the premolars and first molar areas, and very increased overbite (Fig 8). The molars’ crowns were tipped mesially and, due to the excessive overbite, all lateral movements had major interference of the incisors. 

**Figure 9:**
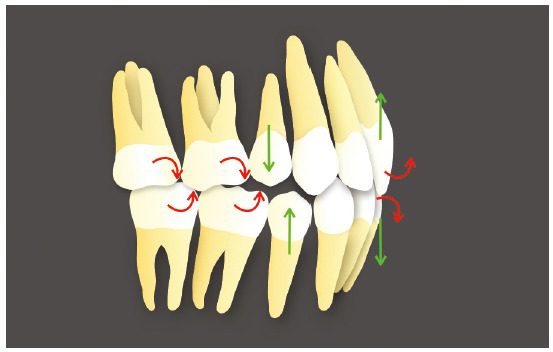
Force system planned for the second set of aligners.

A new set of 37 aligners was planned, for maxillary and mandibular incisors intrusion, premolar extrusion, correction of the molars crown tipping, lower midline correction to the right and mesialization of the left posterior mandibular segment. Cuts for Class II elastic on the left side were made on the six last aligners to help lower midline correction and improve the molars and canines relation. The force system planned for this set is represented on Figure 9. The patient, although very compliant with the use of the aligners, did not use the elastics as recommended. 

**Figure 10:**
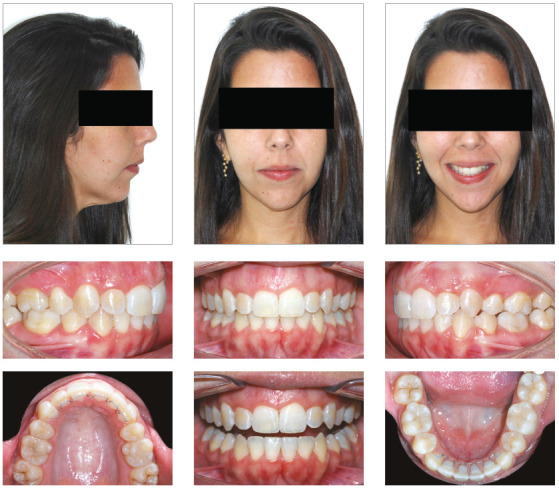
Final photographs of the patient, after the second set of aligners.

The final result showed good occlusal relations, with good root parallelism, 50% overbite, complete closure of the overjet and complete space closure. Despite the incisors intrusion did not happen completely as planned, the protrusive and lateral movements guidances were correctly established, as can be verified in Figure 10. This case showed good finishing parameters and was presented and approved by the Brazilian Board of Orthodontics. 

**Figure 11:**
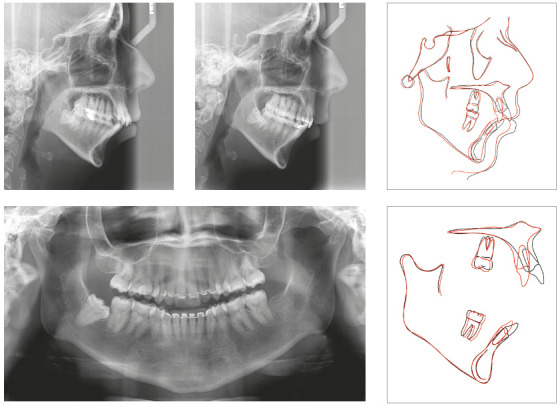
Final panoramic and cephalometric radiographs with superimpositions.

Going through cephalometric superimpositions, we can verify that there was no mesial movement of the maxillary posterior segment, but a slight mesial movement of the mandibular molars. The incisors became more vertical and the mandibular incisors were intruded. The compensatory retraction of the maxillary incisors caused a relative extrusion, due the lingual inclination of the crowns, as expected (Fig 11).

### C) Use of auxiliary mechanics

The aligners systems alone still need further developments to treat more complex malocclusions, such as extractions cases.^18^ That is why the auxiliary resources are so important. As an example, we can take the use of skeletal anchorage on cases where you cannot afford to have any mesial movement of molars. It would make the outcome much more predictable. Miniscrews or miniplates would favor the distalization of the anterior segment without any anchorage loss, having the option of using power arms on the canines to better control the moment of force created, and reduce the undesired effects on the anterior segment.

The use of intermaxillary elastics can also provide more control during space closure. On cases where you have a good mandibular arch but extractions are needed on the maxilla, the use of Class II elastics supported on the mandibular first molars and on the maxillary canines during their distal movement can be an excellent alternative to reduce the bow arch effect. The vertical component of force generated by the elastic would help control the tendency of intrusion in the middle section of the arch. The use of elastics can be started at any point during the treatment, but, if it’s present during the distalization of the canines, we have the advantage of force component that pulls the tooth towards the aligner, making it harder to lose tracking. Similar to what happens on fixed appliances treatments, the intermaxillary elastics can also be used for better anchorage control, avoiding mesial movement of the posterior teeth.

On space closure between teeth with divergent roots, or in the need for uprighting an inclined tooth, the power arms can be an excellent alternative, since they will balance the moments generated by the aligners. OnFigure 12, a case of space closure after a surgical-assisted rapid palatal expansion (SARPE). One can notice that, in spite of the presence of attachments meant to control the root movement, the space closure between the central incisors was happening mainly by mesial inclination of the crowns, causing massive tracking loss of the aligners. In order to revert this situation, power arms made of stainless steel 0.020-in wires were bonded on the lingual surface of the central incisors and a cut was made on the aligners. These power arms were divergent and were activated by a chain elastic pulling them together. Since the power arms raised above the center of resistance of the incisors, it created compensatory moments, contrary to the ones created by the aligners, who were inclining the teeth mesially. It corrected the excessive inclination of the incisors and allowed a more controlled space closure.

**Figure 12:**
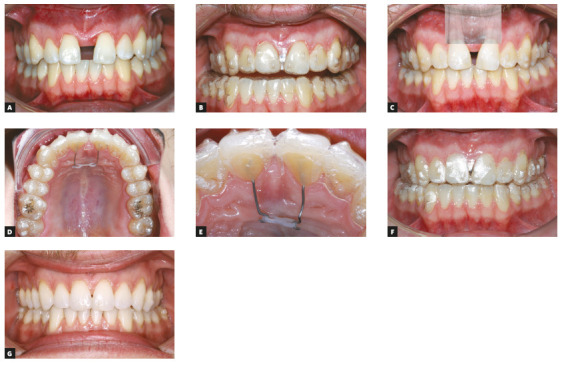
A) Patient after SARPE, starting treatment with aligners. B) Tracking loss due to excessive mesial inclination of the crowns of the incisors. C) Divergent roots. D) Power arms placed to be used along with the aligners. E) Detail of active power arms. F) Improvement of tracking. G) By the end of the aligner set (a new set of aligners was planned then for better finishing).

As discussed earlier, the virtual setup will allow us to analyze the force system step by step and will help us draw a clear map of the biomechanics applied to each step of the treatment. On cases with great incisor retraction, at the end of the planning, the setup will display an anterior open bite, with proclined incisors and molars with distal tipping. These overcorrections are important to be added but, by adding them, the final setup won’t be a reflection of the final occlusion planned by the orthodontist, which may raise, if the orthodontist decide to show this setup to the patient, some doubts and insecurities related to treatment outcomes. If the orthodontist or the patient thinks it is imperative to see the final outcome planned, it can be a good solution to create an ideal setup, with the final occlusion planned just to explain to the patient the treatment objectives and promote better communication and understanding.

## 2 - CASES WITH RECIPROCAL SPACE CLOSURE

On cases where some mesialization of the posterior segment simultaneous with the anterior retraction is desirable, the movements in the sagittal plane work synergistically, meaning that the reciprocal anchorage would tend to favor both movements, optimizing the treatment. Even though this is true, there is also the risk of some of the side effects are potentiated due to the high elasticity of the aligners.

The moment of force created by the mesialization of the molars tends to cause intrusion of their mesial cusps, with consequent mesial inclination of the crowns. The effect on incisors is also similar to the described earlier for maximum anchorage cases. Being so, the same precautions can be made with some minor adjustments.

### Clinical case 2

**Figure 13:**
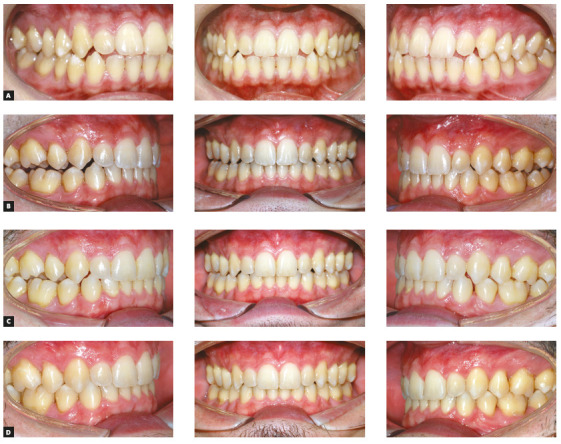
Clinical evolution of a reciprocal space closure, from initial condition (A); to the end of the first set of 51 aligners (B); after the second set of 16 aligners (C); and the final result, after the last sequence of 21 aligners.

Figure 13 displays a case where, specially on the maxillary arch, the movement was made in a reciprocal way. The patient showed Class III molar relationship, anterior open bite, spacing and biprotrusion, being these last two his main complaints (Fig 13A). For the treatment, it was necessary three aligner sequences. The first set had 51 pairs of aligners, where only vertical attachments on canines and premolars were used to avoid crown tipping during the space closure. Since the staging of the movements was not properly done, the side effects discussed earlier were present and intense, especially with molars and premolars inclination, deep bite, extrusion, and lingual inclination of the incisors. On the mandibular arch, where there was more anterior retraction, the negative effects on the incisors were more evident. On the maxillary arch, since there was more mesialization of molars to correct the molar relationship, their inclination and intrusion was much more noticeable, which can be seen in Figure 13B.

A second sequence of 16 pairs of aligners was ordered to correct these problems and improve the case finishing. By that time, the provider released optimized attachments for root control, such as the ones described in Figure 4C, for root control, so it was decided to make an attempt of correcting the teeth inclinations with these attachments, instead of the vertical ones used in the first set.

**Figure 14:**
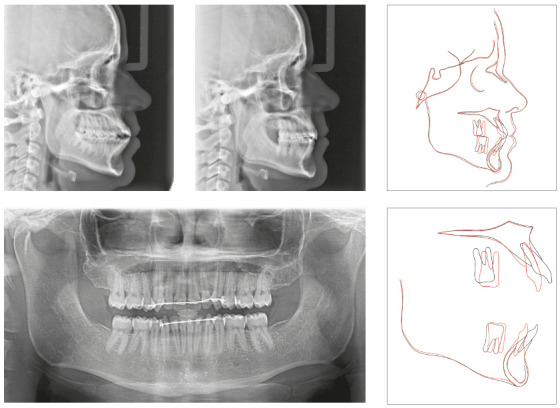
Final panoramic, initial and final radiographs, with cephalometric superimpositions.

The results were not satisfactory, as it can be seen on Figure 13C, and a third set with 21 pairs of aligners was ordered, but this time with cuts for buttons bonded on the first molars for intermaxillary elastic use simultaneously with the aligners. The use of vertical elastics was kept for 45 days after the removal of the aligners for better settling of the occlusion, after which, treatment was finished with proper molar relationship and better inclination of incisors. The overbite was still deeper than the ideal, even though there was no interference with lateral or protrusive disocclusion guidances. Figure 14 displays the initial and final cephalometric radiographs and superimpositions.

Further improvement of the deep bite would demand a new set of aligners, which was refused by the patient, claiming to be completely satisfied with that result, both esthetically and functionally. This situation is common during the learning curve with aligners and makes it very clear why the professional must have a deep knowledge of the aligners biomechanics, the effects of its resources and be much more proactive during the treatment planning and anticipate the possible side effects of the chosen biomechanics. If the orthodontist waits until the end of the aligners set to see what went down different from planned, and only then take corrective actions, this will make treatments with high risks of side effects much longer. If we consider that aligners treatments are completely reliant on patient compliance, in longer treatments we risk having this cooperation worn out and the compliance reduced.

When used only as anchorage units, the molars will already tend to incline mesially during retraction of canines and incisors. On cases where there will be a force to move molars mesially, this tendency will be even bigger. That is why it is recommended some distal inclination to the crown of these molars, similar to the tip-back bends made on anchorage on Tweed-Merrifield technique. A six-degree distal inclination on the molars was suggested to compensate this tendency.^20^ By doing this, notice that the aligner will tend to disadapt on the mesial cusp, so it is important to increase the retention of the aligner by adding an attachment on the mesial cusp or bonding buttons to use vertical elastics. These precautions will make it more likely that the aligner keeps the tracking the whole movement.

If we consider the bow arch effects of the aligners and the root volume of the molars, the chance of having heavy side effects of lingual inclination and extrusion of the incisors is even bigger. To compensate for that, buccal crown inclination of 10 degrees must be added as an overcorrection, as well as a marked intrusion on the incisors.^20^ In case it is necessary to compensate exclusively by adding buccal inclination, if, for some reason it is not possible to add intrusion on incisors, it will be made by adding pressure areas on the cervical buccal surface and on the incisal lingual surface. This force arrangement may cause the aligner to disadapt, so, to avoid that, it is suggested that a retention attachment is added at the buccal surface. It will prevent the aligner from disadapting and the movement will be better expressed.

It has been suggested that several movements need to be overcorrected when planning aligner treatments, considering that not every movement planned will be expressed to the full extent. Some movements have been verified to respond from 28 to 56%, with an average of 50% of what was planned.^21^

A good strategy related to overcorrections of some isolated movements, such as rotations or intrusions, is to ask that the movement is done normally during the aligner sequence, but the overcorrection to be done alone, at the end of the sequence. This way, if the initially planned movement is enough and the overcorrection is not needed, the professional can just skip these aligners and interrupt the treatment sooner. It is important though not to skip overcorrections in movements that tend to be unstable, with high risk of relapse, because it will be important for long term stability, even if the tooth respond well.

In cases of reciprocal space closure, the use of other auxiliary resources may be less critical if skeletal anchorage is associated with the aligners, but can still be very helpful in the prevention of side effects. Power arms, for example, can improve dramatically the root control and keep root parallelism during distalization of the canines and mesialization of molars. Despite moments created using vertical or optimized attachments to this end, it was clear on Figures 9 and 13 that these resources alone may not be enough. Vertical intermaxillary elastics on the medium segment of the arch on buttons bonded to the teeth may be a more predictable strategy to reduce the bow arch effect. The use of box elastics, even after the removal of the aligners, helps improve the final settling of the occlusion, compensating these inclination effects on molars with its extrusive force components caused by the elastics.

## CONCLUSION

It is possible to treat complex cases with aligners. However, to obtain good aesthetic and functional results, it is necessary that the orthodontist:

» Select the patient's degree of motivation and collaborative profile.
» Invest a good amount of time in training, to better understand the characteristics of the appliances and the limitations of the technique.
» Prepare an individualized planning, having full awareness and control of the forces to be applied; anticipate and implement mechanisms to control their side effects.
» Consider the need to use auxiliary resources and overcorrections to address deficiencies in the aligner systems.

Digital planning and the use of aligners can be great allies for orthodontists, as well as can induce them to prescribe very unpredictable movements, since the virtual environment does not necessarily reflect *in vivo* conditions.
